# The Womb Cancer Awareness Measure (WCAM): development of an instrument to assess public awareness of endometrial cancer

**DOI:** 10.1136/ijgc-2023-004796

**Published:** 2023-12-06

**Authors:** Olivia Jones, Hannah Young, Helen Clarke, Emma J Crosbie, Vanitha N Sivalingam

**Affiliations:** 1 Division of Cancer Sciences, The University of Manchester, Manchester, UK; 2 Department of Colposcopy and Gynaecological Oncology, St Mary's Hospital, Manchester University NHS Foundation Trust, Manchester, UK; 3 Gynaecology, Liverpool Women's Hospital NHS Foundation Trust, Liverpool, UK

**Keywords:** Endometrial Neoplasms, Endometrium

## Abstract

**Objective:**

To develop and test a novel instrument to assess public awareness of endometrial cancer symptoms and risk factors in a UK population.

**Methods:**

A 36-item questionnaire was developed through literature review and extraction from cancer awareness materials. The Womb Cancer Awareness Measure (WCAM) was tested for content validity in 65 self-identified female research participants and 10 endometrial cancer experts prior to UK-wide field testing using social media. Test-retest reliability was assessed over 2 weeks, construct validity was assessed by comparing womb cancer experts and non-medical academics, and sensitivity to change was assessed by comparing scores of participants who read an endometrial cancer leaflet with those given a control leaflet.

**Results:**

Fifty-two percent of the items in the test-retest reliability showed >80% agreement. Construct validity was demonstrated; endometrial cancer experts achieved higher scores (median 79 (IQR 18)) than non-medical academics (median 50 (IQR 18)) (p<0.001). The WCAM was sensitive to change; volunteers who read an endometrial cancer leaflet showed greater awareness (median 73 (IQR 9)) than those who read the control leaflet (median 59 (IQR 9)) (p<0.001). Knowledge of endometrial cancer red flag symptoms and risk factors was poor in the 847 UK-based participants.

**Conclusions:**

Our findings support the validity and reliability of the Womb Cancer Awareness Measure in assessing public awareness of endometrial cancer. In a UK population sample, knowledge of warning symptoms and risk factors was low, highlighting the need for public awareness campaigns.

**WHAT IS ALREADY KNOWN ON THIS TOPIC**

- Increased awareness of risk factors and warning symptoms of endometrial cancer could encourage personal risk perception, modify health behaviors and prompt early presentation.

**WHAT THIS STUDY ADDS**

- In a well-educated UK female population, only 13% and 25%, respectively, were able to identify >2 risk factors or warning symptoms for endometrial cancer, with almost half lacking confidence in the detection of red flag symptoms.

**HOW THIS STUDY MIGHT AFFECT RESEARCH, PRACTICE OR POLICY**

- The Womb Cancer Awareness Measure can be used to measure effectiveness of endometrial cancer public awareness initiatives, one of the most important research priorities in endometrial cancer.

## Introduction

Endometrial cancer is the sixth most common cancer occurring in women worldwide, with more than 417 000 new cases being diagnosed in 2020.^1^ The incidence rates are highest in North America and Europe, with increasing incidence being reported in countries with rapid socioeconomic transition.^2^ In the UK, endometrial cancer is the most common gynecological cancer with a 55% increase in the incidence rate since the 1990s.^3^ The increased incidence of endometrial cancer is attributed to the prevalence of risk factors,^2^ especially obesity.^4^ One-third of endometrial cancer cases could be preventable,^2 5^ supporting the need for targeted prevention strategies, while non-modifiable risk factors could be addressed by increased public awareness, risk perception, and encouragement of positive health behaviours.^6^

Early-stage diagnosis of cancer increases survival, reduces treatment morbidity, and improves quality of life. Lung and bowel cancer symptom awareness campaigns have improved the likelihood of early presentation, with an increase in healthcare attendance with publicized symptoms.^7^ In the absence of endometrial cancer screening programs, warning symptom knowledge could trigger rapid presentation and diagnosis.^6 8^ Some women attribute warning symptoms including abnormal vaginal bleeding and discharge^9^ to benign causes and avoid seeking help due to competing family and work demands, difficulty accessing healthcare, and fear of wasting doctors’ time.^10 11^ In a German study, poor awareness of endometrial cancer risk factors was identified, demonstrating a need for risk awareness-raising initiatives.^12^

The UK Government’s Cancer Reform Strategy identified the need for standardized measurements of cancer awareness to promote earlier diagnosis and improve survival rates,^13^ resulting in the development of the Cancer Research UK (CRUK)’s Cancer Awareness Measure.^14^ Identifying effective ways of raising public awareness about endometrial cancer is a top 10 research question for patients and clinicians.^15^ The Womb Cancer Awareness Measure (WCAM) was developed and tested as a self-complete instrument to assess public awareness of endometrial cancer, consisting of items related to knowledge of red flag symptoms and risk factors (see online supplemental appendix S1). The assessments of the psychometric properties of the instrument and the first survey of endometrial cancer awareness in the UK are reported.

10.1136/ijgc-2023-004796.supp1 Supplementary data

## Methods

The study was designed in two phases: (1) development and content validity of the WCAM in research participants and womb cancer experts to ascertain validity, reliability, and responsiveness; and (2) testing of the WCAM in a cohort of UK participants.

### Questionnaire Development, Validation, and Scoring

Based on a priority setting partnership with patients and healthcare professionals, ‘womb cancer’ is the preferred lay phrase to describe endometrial cancer.^15^ Eligible items were identified from the literature and public-facing cancer information websites including cancerresearchuk.org and macmillan.org.uk.

Items assessed the ability to detect warning symptoms and personal risk to reduce patient-attributable delays (see online supplemental table S1). An unprompted question was followed by a prompted checklist of endometrial cancer symptoms and risk factors in addition to distractor items.^14^ Questions were designed to evaluate confidence levels in detecting a red flag symptom and the rapidity of seeking medical care, modeled on existing instruments for breast,^16^ ovarian, and cervical cancer.^17^

The first version was circulated to a panel of scientists, gynecological oncologists, and patient representatives; irrelevant and ambiguous items were removed. Participants were invited to comment on the content and design of the instrument (see online supplemental appendix S1). Two independent observers scored each completed WCAM according to a proforma (see online supplemental appendix S2). Higher scores indicated greater endometrial cancer awareness.

### Content Validity Studies

Research participants in the test-retest reliability and sensitivity to change analyses (n=65) comprised university staff and students who self-identified as biologically female (online supplemental table S2) and who responded to university-based physical and electronic advertisements. Womb cancer experts (n=10) and non-clinical scientists (n=16) in the construct validity analysis comprised both male and female participants recruited through electronic advertisements, university distribution lists, and the researchers’ networks. Statistical analyses were performed using SPSS v25.0. A p value of <0.05 was regarded as statistically significant.

### Readability

Readability was calculated using the Flesch Reading Ease formula (Microsoft Word v.2015). Scores ranged from 0 to 100, with higher scores indicating a text that is easy to read. A score of >60 is considered acceptable readability for the average adult.^18^

### Test-Retest Reliability

To establish consistency, participants completed it twice within 14 days as baseline knowledge is likely to remain constant and original answers are not recalled during this timeframe.^14^ Test-retest reliability was assessed for each item using Cohen’s kappa coefficient.^16^ Large positive kappa values (range −1 to +1) represent high levels of agreement. The percentage of exact agreements for each item was calculated as the kappa statistic can be distorted or indeterminate if variables are nearly or completely constant.^19^

### Construct Validity

The ‘known-groups’ method was used to assess whether items could measure the construct of endometrial cancer awareness.^20^ Differences in correct WCAM responses from gynecological oncologists (n=10) and non-medical academics (n=16) were tested using Mann–Whitney U and χ^2^ tests. Validity is established when significant differences in knowledge scores between two groups known to differ in cancer awareness levels are detected.

### Sensitivity to Change

Participants were randomized to read one of two leaflets before completing the WCAM. The intervention group (n=22) read an endometrial cancer leaflet while the control group (n=21) received a leaflet of similar length and readability about climate change.

### Testing the WCAM in the UK Population

The validated WCAM was tested in a UK population sample between May and July 2021 to measure public awareness of endometrial cancer and barriers to diagnosis. Advertisements on social media platforms invited participants aged ≥18 years who self-identified as biologically female to participate. To diversify sampling, the survey was shared on the researchers’ community groups and retweeting was encouraged to enable a snowball effect.^21^ Dissemination through social media pages of patient support groups (endometrial cancer, polycystic ovary syndrome) gained responses from participants previously at risk or at increased risk of endometrial cancer.

We compared the prompted and unprompted knowledge scores of red flag symptoms to assess the educational effect of the checklist. Knowledge scores in participants with experience of endometrial cancer were compared with those with no previous experience. Prior endometrial cancer experience was determined by self-declaration of occupation (healthcare professional) and the question “Have you, a relative, or close friend ever been diagnosed with womb cancer?”

## Results

The Flesch Reading score of 71 demonstrated a reading standard that is acceptable and easier to understand than average adult reading material.

### Test-Retest Reliability

The characteristics of the participants in the validation studies are shown in online supplemental table S2. The median age was 41 (range 24–65) and 5% were of non-white ethnicity. A total of 22 of 23 (96%) participants completed the WCAM at both time points (Table 1). Thirty-two percent of the kappa statistics were in the range of moderate to substantial agreement (0.41–0.70). Thirty six percent were negative or indeterminate due to participants responding with the same answer in both attempts (‘yes’ for postmenopausal bleeding) as items achieved high agreement (86–100%). Over half the items exceeded 80% agreement with risk factor and relative risk items scoring lower. Seventy-three percent had an improved total knowledge score during a second attempt (mean improvement 5.5±9.3%).

**Table 1:** Test-retest reliability of the Womb Cancer Awareness Measure

	% of exact agreements	Kappa statistic*
Identifying correct warning signs		
Bleeding between periods	100	–
Having heavier periods	73	−0.08
Postmenopausal bleeding	100	–
Bloody discharge	91	0.45
Pelvic pain	95	–
Anemia	82	0.65
Weight loss	68	0.24
Identifying distractor signs		
Pain during sex	91	0.46
Identifying correct risk factors		
Not having children	64	0.27
Starting periods at a young age	77	0.5
Being postmenopausal	64	0.24
Having had a late menopause	73	0.46
Having a close relative with womb cancer	86	–
Having polycystic ovary syndrome	68	0.37
Having diabetes	82	0.65
Being overweight	82	0.65
Having a sedentary lifestyle	86	0.58
Using tamoxifen	55	0.10
Identifying correct distractor factors		
Having a hysterectomy	86	–
Taking the combined contraceptive pill	82	−0.10
Having a negative cervical smear	59	0.36
Knowledge of peak age of incidence		
A 30-, 50-, 70-, or 80-year-old woman	50	0.27
Knowledge of endometrial cancer screening	77	0.54

*The kappa statistic represents the level of agreement and ranges between −1 and +1. Large positive values suggest a higher level of agreement. The percentage agreement shows the percentage of participants who answered each item the same way in repeated attempts.

### Construct Validity

The WCAM discriminated between the knowledge scores of cancer experts (median score 78.5 (IQR 18)) and non-experts (median score 50 (IQR 18)) (p<0.001) (Table 2). Experts listed more unprompted red flag symptoms (median difference 2) and risk factors (median difference 2) (p<0.001) and correctly selected more warning symptoms (median difference 1.5) and risk factors (median difference 3.5) (p=0.041) when prompted.

**Table 2:** Construct validity of the Womb Cancer Awareness Measure

Awareness section	Endometrial cancer experts (n=10)	Non-medical academics (n=16)	Median difference	P value*
	Median (IQR)	Median (IQR)		
Total knowledge score* (max: 98)	78.5 (18)	50 (18)	28.5	<0.001
Unprompted warning signs and symptoms (max: 8)	4 (1)	2 (0)	2	<0.001
Prompted warning signs and symptoms (max: 7)	7 (2)	5.5 (1)	1.5	0.041
Unprompted risk factors (max: 10)	5.5 (3)	2 (2)	2	<0.001
Prompted risk factors (max: 70)	57.5 (6)	47.5 (4)	3.5	<0.001
Peak age of incidence (max: 2)	2 (0)	1 (2)	1	0.001
	**n (%)**	**n (%)**	**χ** ^**2**^	**P value** *
Awareness of screening program (correct)	10 (100)	9 (56)	3.97	0.02

*Assessed with χ^2^ tests.

### Sensitivity to Change

The intervention and control groups in the sensitivity to change analysis were comparable in age, ethnicity, and educational attainments (see online supplemental table S2). Participants who received the cancer information leaflet (intervention) had significantly greater knowledge scores than the controls for all items except in knowledge of warning symptoms (Table 3). Both groups had low unprompted scores for warning symptoms and risk factors. The intervention group’s median scores (74%) were similar to scores achieved by cancer experts (79%), demonstrating the educational effect of patient information leaflets.

**Table 3:** Sensitivity to change: differences in the Womb Cancer Awareness Measure scores between control and intervention patients

Awareness section	Control (n=22)	Intervention (n=21)	Median difference	P value*
	Median (IQR)	Median (IQR)		
Unprompted warning signs and symptoms (max: 8)	2 (2)	2 (1)	0	0.5
Prompted warning signs and symptoms (max: 8)	6 (2)	7 (1)	1	0.155
Unprompted risk factors (max: 10)	1 (1)	4 (3)	3	<0.001
Prompted risk factors (max: 65)	49 (5)	57 (7)	8	<0.001
Age of incidence (max: 2)	1 (2)	2 (1)	1	0.002
Total knowledge score (max: 98)	59 (9)	72.5 (9)	13.5	<0.001
	**n (%)**	**n (%)**	**χ** ^**2**^	P value*
Screening program (correct)	8 (36)	20 (91)	13.9	<0.001

*Assessed with χ^2^ tests.

### Testing Endometrial Cancer Awareness in the UK Population

A total of 847 participants were recruited and, of these, 763 participants (90%) answered all items. The median age was 44 years (range 19–80) and the majority (94%) were White. Seventy percent of participants were educated to degree level or higher. A third of participants had either received a previous endometrial cancer diagnosis themselves or in someone close to them. An additional 11% had a medical condition that predisposed them to endometrial cancer including polycystic ovary syndrome (6%) and endometrial hyperplasia (3%) (see online supplemental table S3).

**Figure 1:**
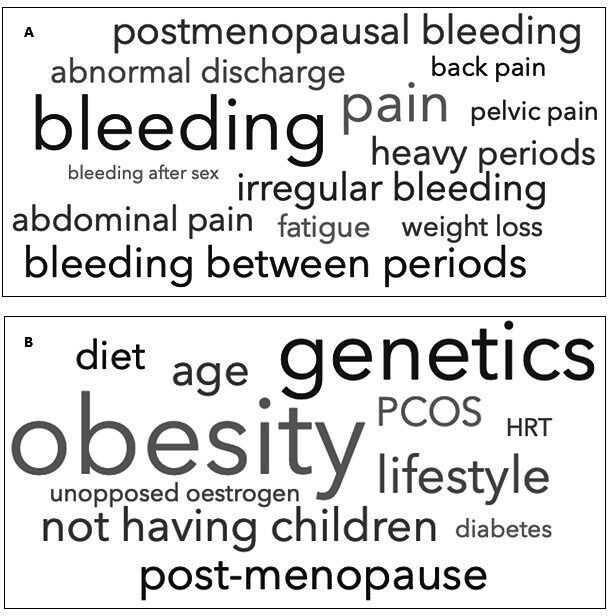
(A) Word cloud representing most frequent correct responses to the unprompted womb cancer warning sign items. (B) Word cloud representing the most frequent correct responses to the unprompted cancer risk factor items. The larger the size of the word, the more frequently it was identified by participants.

Only one-quarter of participants could identify more than two unprompted warning symptoms for endometrial cancer. The word cloud (Figure 1A) depicts the most frequent correct responses; abnormal bleeding was the most recognized symptom. Abnormal discharge, fatigue, and weight loss were reported in 10% of responses. A third of women inaccurately reported bloating as a warning symptom of endometrial cancer. Other common misconceptions included change in bowel habit and dyspareunia (online supplemental figure S1). Only 13% of participants could identify more than two risk factors; the most identified were obesity (43%) and genetics (27%) (Figure 1B).

When prompted, most participants recognized intermenstrual bleeding (90%), pelvic pain (86%), and postmenopausal bleeding (85%) as red flag symptoms (see online supplemental table S4). A genetic predisposition to endometrial cancer (89%) was considered the most significant risk factor, followed by obesity (82%) and a sedentary lifestyle (70%) (Figure 1B). Smoking and the combined contraceptive pill were incorrectly considered risk factors by a majority of participants (95% and 85%, respectively) (see online supplemental figure S2). Over 70% recognized that a hysterectomy protects against endometrial cancer. Most participants (85%) did not recognize that older women (ie, ≥70 years) are at greatest risk of endometrial cancer and over 50% of participants incorrectly thought that an endometrial cancer screening program exists in the UK. Almost half of the participants were ‘not at all confident’ that they would be able to detect a red flag symptom, although 55% said they would seek help immediately if a symptom was observed. Of the 28% who said they would wait longer than a month before seeking help, many cited worries about being perceived as complainers and dismissed by doctors. Concerns about difficulties accessing GP appointments due to COVID-19 restrictions were frequently reported.

Participants without prior experience of endometrial cancer had the lowest mean total knowledge (58%), while the physicians had the highest (69%) (online supplemental table S5).

## Discussion

### Summary of Main Results

The WCAM achieved high completion rates (90%) in a population of people at risk of endometrial cancer and may prove a useful tool to measure the effect of public awareness initiatives. Improved scores in repeat completion of the WCAM in test-retest reliability demonstrate the ‘mere measurement’ effect, where completion of a questionnaire can improve awareness.^22^ Construct validity was established as experts achieved consistently higher knowledge scores than non-medical academics.

The WCAM was tested on the UK population by targeting people who identified as biologically female and thus at risk of developing endometrial cancer. Our sample was enriched with participants with previous endometrial cancer experience (1/3) who achieved greater total knowledge scores compared with the general public. As only 3% of the UK population will be diagnosed with endometrial cancer, public awareness is likely even lower than presented.

### Results in the Context of the Current literature

Awareness of red flag symptoms was low among the participants; in the absence of prompts, only 24% could identify more than two symptoms. While abnormal bleeding was recognized by 35% as a warning symptom, few were able to characterize the bleeding (eg, post-menopausal), which may impair communication during telephone consultations. Almost one-third of patients would delay seeking help for symptoms; women with gynecological cancer sometimes normalize their symptoms and will only seek help if they perceive them as serious.^11^

There was a poor awareness of risk factors, with the risks posed by reproductive factors (nulliparity, early menarche, late menopause) not being recognized. Genetic predisposition was considered the most significant risk factor, in keeping with earlier findings in German women.^12^ In our study, obesity was a recognized risk factor which may be attributed to the 2018–19 CRUK campaign targeting the modifiable role of obesity in cancer.^23^

Protective factors associated with endometrial cancer, such as smoking and the combined contraceptive pill, were largely unrecognized; in fact, almost all participants thought these were risk factors.^24 25^ Smoking cessation public health campaigns are the likely cause for participants thinking smoking causes all cancers. Participants could be confused with increased risk of breast cancer with the hormonal contraception, or may be fearful of long-term hormonal treatments.^26^ Multiple sexual partners and sexually transmitted infections (human papillomavirus) were mistakenly thought to increase the risk of endometrial cancer. A prior study found that one in five women associated gynecological cancer with sexual promiscuity, which prevented them from seeking help.^27^ Confusion about the risk factors and symptoms of different gynecological cancers suggests a role for a pan-gynecological cancer awareness campaign to help the public recognize different symptoms associated with gynecological cancers.

### Strengths and Weaknesses

In addition to the robust validation studies and stakeholder input, a strength of this study is the assessment of endometrial cancer awareness in a large UK population sample. A social media approach was necessary due to COVID-19 restrictions; however, this approach had limitations. There was a lack of diversity within the population (6% were non-white); white ethnicity predicts greater awareness of general cancer symptoms.^28^ Non-white women are more likely to experience diagnostic delay and have worse outcomes when diagnosed with breast cancer.^29^ In the USA, incidence rates of endometrial cancer have risen most in non-Hispanic Black and Asian women. Furthermore, the 5-year relative survival is significantly worse in non-Hispanic Black women compared with White or Asian women.^30^ The high education level among participants further limits generalizability, as currently only one-third of the UK population aged 16 and above are educated to degree level.^31^ Endometrial cancer mortality rates are highest among women of low socioeconomic status,^32^ so further data on endometrial cancer awareness according to sociodemographic index are needed to evaluate the need for specific targeting of at-risk populations.

As the WCAM was publicized through social media, the participants are digitally-aware people. It is likely that a proportion of people aged ≥70 years, at greatest risk of endometrial cancer, were excluded from participating. Only 21% of people aged >75 years have a social media profile compared with 93% of people aged 25–34 years.^33^ Our survey specifically targeted those who self-identify as biologically female (ie, born with a womb), so we do not know if gaps in knowledge are even greater in people who are biologically male. The main aim, however, was to assess the awareness in those at greatest risk of developing endometrial cancer (ie, biologically female with an intact uterus) as a means of encouraging early presentation.

### Implications for Future Practice

The first evaluation of endometrial cancer awareness in the UK shows low confidence in symptom detection and inaccurate knowledge about risk factors, demonstrating a need for high quality public awareness campaigns.^34^ Similar to successful breast, bowel, lung, and ovarian cancer awareness campaigns, an endometrial cancer awareness program should aim to raise awareness of signs and symptoms and increase early presentation to aid early diagnosis. Future work with the WCAM should include paper-based implementation and targeted testing in older patients, non-white ethnic groups (with translated versions), lower socioeconomic populations, and those with lower educational attainment to sample all people irrespective of their level of engagement with technology. Future assessment of the WCAM will require data on gender, sexual identity, and disability to provide additional validity and generalizability. These further studies are key research priorities in detecting cancer early and may address any cultural, religious, or gender-driven issues which may prevent recognition and reporting of symptoms of endometrial cancer.^35^

## Conclusion

The WCAM is shown to be a discriminate and reliable tool to assess public awareness of endometrial cancer. Knowledge of signs and symptoms of endometrial cancer was deficient in a UK sample, illustrating the need for appropriately designed and targeted public awareness campaigns.

## Data Availability

Data are available upon reasonable request. In accordance with the journal’s guidelines, we will provide our data for independent analysis by a selected team by the Editorial Team for the purposes of additional data analysis or for the reproducibility of this study in other centres if requested.
