# Supplementary material for: The Womb Cancer Awareness Measure (WCAM): development of an instrument to assess public awareness of endometrial cancer
Source: Int J Gynecol Cancer. 2023 Dec 6;34(1):73–9. doi: 10.1136/ijgc-2023-004796 (PMC10850626; doi:10.1136/ijgc-2023-004796)
Supplement: Supplementary data [file ijgc-2023-004796supp001.pdf]

The Womb Cancer Awareness Measure (WCAM): Development of a validated tool to assess public awareness of endometrial cancer

Supplementary Tables , Figures and Appendices

Table S1 Summary of items included in the Womb Cancer Awareness Measure validation studies

Awareness section	Items included in Womb CAM	Maximum score
**Warning signs and symptoms**	1 open item<br>8 prompted items	8<br>8
**Risk factors**	1 open item<br>13 prompted items	10<br>65
**Peak age of incidence**	1 item	2
**Endometrial Cancer screening**	1 item	1

Table S2 Sociodemographic characteristics of participants (n = 65) in the initial validation studies

	**Test-retest reliability (*n*=22)**	**Construct validity (*n*=26)**		**Sensitivity to change (*n*=43)**	
		**Experts (*n*=10)**	**Non-medical academics (*n*=16)**	**Control (*n*=21)**	**Intervention (*n*=22)**
**Age, median (range)**	41 (24-65)	46.5 (30-57)	41.5 (20-65)	23 (20-61)	22 (19-58)
**Education level, *n* (%)**					
**Secondary school (GCSE/ A Levels)**	2 (9)	-	2 (13)	9 (43)	12 (55)
**Undergraduate**	13 (59)	1 (10)	8 (50)	7 (33)	9 (41)
**Postgraduate**	6 (28)	9 (90)	5 (32)	5 (24)	1 (5)
**Other**	1 (5)	-	1 (6)	-	-
**Ethnicity, *n* (%)**					
**White British or Other White Ethnicity**	21 (95)	9 (90)	16 (100)	16 (76)	19 (86)
**Black Ethnicity**	-	-	-	-	1 (5)
**Asian**	-	1 (10)	-	5 (24)	1 (5)
**Arab**	-	-	-	-	1 (5)
**Mixed/Multiple ethnic groups**	1 (5)	-	-	-	-

Table S3 Sociodemographic characteristics of participants in the Validation Study (UK Population Sample)

	**UK population sample, *n* (%) (*n* = 847)**
**Age, median (range) , years**	44 (19-80)
By age brackets, years	
18-24	50 (6.3)
25-34	139 (17.4)
35-44	239 (30.0)
45-54	229 (28.7)
55-64	140 (17.6)
>65	50 (6.3)
**Ethnicity, *n* (%)**	
White British/Other	800 (94.0)
Black African/Caribbean/ British	6 (1.0)
Asian	22 (3.0)
Arab	1 (0.1)
Mixed/Multiple ethnic groups	17 (2.0)
Other	3 (0.5)
**Education level, *n* (%)**	
No educational qualifications	21 (3.0)
GCSEs and A Levels or equivalent	229 (27.0)
Undergraduate degree	288 (34.0)
Postgraduate degree	303 (36.0)
Other	7 (1.0)
**Previous womb cancer diagnosis*, *n* (%)**	266 (31.02)
**Predisposing medical condition, *n* (%)**	
Polycystic ovary syndrome	51 (6.0)
Endometrial hyperplasia	27 (3.2)
Type 2 diabetes	15 (1.7)
Metabolic syndrome	4 (0.6)

*(either themselves or someone close to them)

Table S4 Prompted responses to womb cancer symptoms and risk factors

**Question**		**Identified as risk factor, *n* (%)**
**Endometrial cancer symptoms**	Intermenstrual bleeding	744 (90)
**(*n* = 824)**	Pelvic pain	711 (86)
	Post-menopausal bleeding	704 (85)
	Heavier periods	589 (72)
	Pain during sex[ϕ]	577 (70)
	Abnormal discharge	542 (66)
	Weight loss	537 (65)
	Anaemia	347 (58)
**Endometrial cancer risk factors***	Smoking[ϕ]	757 (95)
**(*n* = 798)**	Family history	709 (89)
	Combined oral contraceptive pill[ϕ]	677 (85)
	Being overweight	654 (82)
	Sedentary lifestyle	558 (70)
	PCOS	472 (59)
	Postmenopausal	458 (57)
	Diabetes	397 (50)
	Negative cervical smear[ϕ]	373 (47)
	Nulliparity	302 (38)
	Early menarche	287 (36)
	Tamoxifen	288 (36)
	Late menopause	207 (26)
	Hysterectomy[ϕ]	181 (23)

*For risk factors, 'yes'=score of >4 (agree/strongly agree), 'no'=score of ≤3 (neutral/ disagree/ strongly disagree)

[ϕ]Distractor item

Table S5 Total knowledge scores of participants in general population sample

	No prior experience (*n* = 519)	Endometrial cancer experience* (*n* = 224)	Physicians (*n* = 24)
**Total knowledge score, mean (SD)<br>Max: 98**	57 (7.2)	59 (8.0)	68 (8.0)
**One way-Anova comparing three groups**		<0.001	<0.001
**One way Anova comparing two groups**			<0.001

*Participants who answered ‘yes’ the question ‘Have you, a relative, or close friend ever been diagnosed with womb cancer?’ p-values <0.05 are regarded as statistically significant.

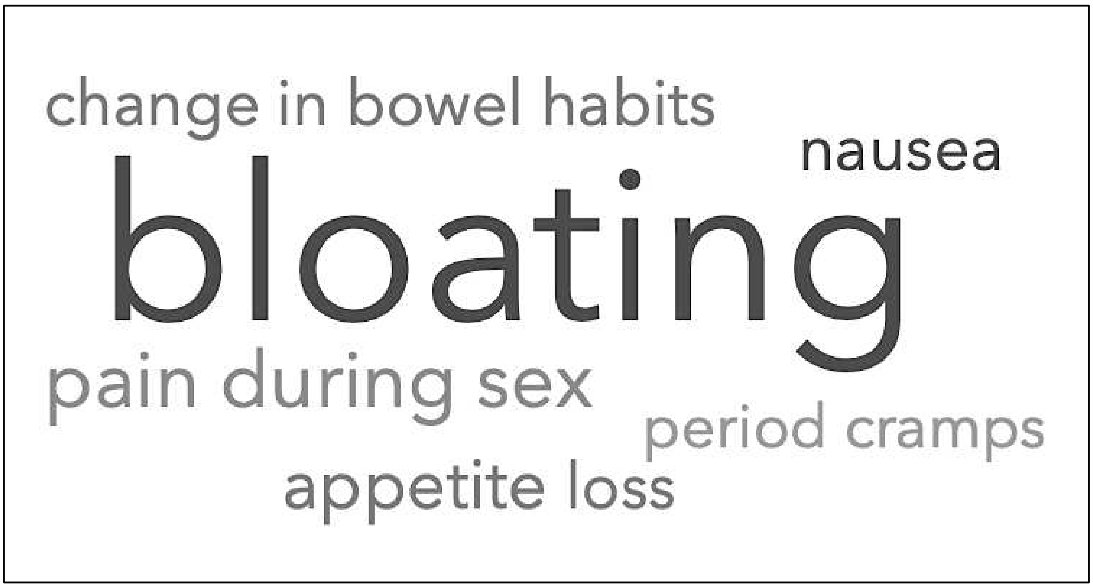

Figure S1 Word cloud representing common misconceptions about the symptoms of endometrial cancer warning signs based on unprompted responses.

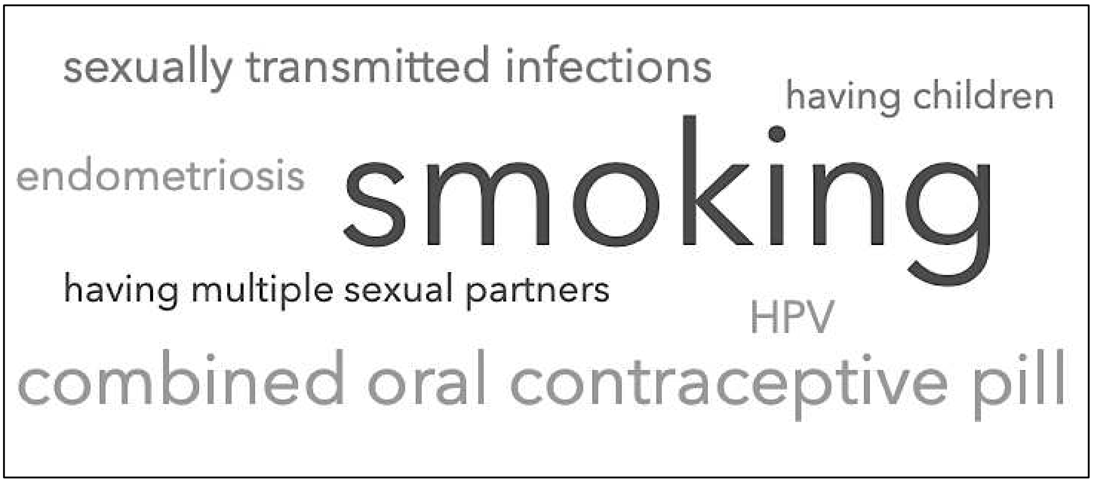

Figure S2 Word cloud representing common misconceptions about the risk factors for endometrial cancer based on unprompted responses

Appendix S1 The Womb Cancer Awareness Measure

Part 1: Firstly, we would like to know a little about you. This information helps us to build a picture of your background and analyse the results.

1. What age are you now?

   ☐ years old

2. Which of the following describes the highest level of education you achieved?

☐ No qualifications<br>☐ CSE/GCSE/O-Levels/Intermediate GNVQ<br>☐ A levels/ Advanced GNVQ/BTEC National Diploma<br>☐ Degree level e.g., BA, BSc and/or Higher National Diploma	☐ Postgraduate qualification e.g., Postgraduate Diploma or Master's Degree<br>☐ Doctorate/PhD<br>☐ Other, please specify : ☐

3. What is your current occupation (or most recent occupation, if you are retired)?

____

4. How would you describe yourself? (Please select **one**)

White:

- ☐ English/Welsh/Scottish/Northern Irish/ British
- ☐ Irish
- ☐ Other White background, please specify: ☐

Black/African/Caribbean/Black British

- ☐ African
- ☐ Caribbean
- ☐ Any other Black/African/Caribbean background, please specify: ☐

Asian:

- ☐ Indian
- ☐ Pakistani
- ☐ Bangladeshi
- ☐ Chinese
- ☐ Any other Asian background, please specify: ☐

Mixed/multiple ethnic groups:

- ☐ White and Black Caribbean
- ☐ White and Black African
- ☐ White and Asian
- ☐ Any other Mixed/ multiple ethnic background, please specify: ☐

- ☐ Arab
- ☐ Any other ethnic background, please specify: ☐

5. What is your current height and weight?

Height ____ft ___ inches OR _____ cm **Weight** ______stone OR _____lbs OR ______kg

6. Have you or a relative/close friend ever been diagnosed with womb cancer?

Yes ☐ No ☐

7. Have you ever been diagnosed with any of the following conditions? (Please select **ALL** that apply)

Polycystic ovary syndrome ☐

Type 2 Diabetes ☐

Metabolic Syndrome ☐

Endometrial hyperplasia (thickening of the womb lining) ☐

Part 2: Womb Cancer Awareness Measure

**6. There are several warning symptoms and symptoms of womb cancer. Name as many as you can think of:**

**7. Some of the following could be symptoms of womb cancer. Tell us which you think are SYMPTOMS OF WOMB CANCER:**			
	Yes	No	Don't know
Bleeding in between your periods?	☐	☐	☐
Having heavier periods than normal?	☐	☐	☐
Vaginal bleeding after the menopause?	☐	☐	☐
Bloody or pink and watery vaginal discharge?	☐	☐	☐
Pain or discomfort in the pelvic area?	☐	☐	☐
Pain during sex?	☐	☐	☐
Having a low blood or iron count (i.e., anaemia)?	☐	☐	☐
Weight loss?	☐	☐	☐

**8. If you had a symptom you thought could be a sign of womb cancer, how soon would you make an appointment with your doctor about it?**

**9. In the next year, who is most likely to develop womb cancer?**	
A woman aged 30 years	☐
A woman aged 50 years	☐
A woman aged 70 years	☐
A woman aged over 80 years	☐
Womb cancer is not related to age	☐

**10. What things do you think increase a woman's chance of developing womb cancer?**

**11. There are some things that can increase a woman's chance of developing womb cancer. How much do you agree that each of these things can INCREASE the risk of developing womb cancer?**					
	**Decreases Risk**			**Increases Risk**	
	Strongly disagree	Disagree	Neutral	Agree	Strongly Agree
Not having children	☐	☐	☐	☐	☐
Starting periods at a young age	☐	☐	☐	☐	☐
Having gone through the menopause	☐	☐	☐	☐	☐
Having a late menopause	☐	☐	☐	☐	☐
Having a close relative with womb cancer	☐	☐	☐	☐	☐
Having had a hysterectomy (womb removed)	☐	☐	☐	☐	☐
Having a negative cervical smear test	☐	☐	☐	☐	☐
Having polycystic ovary syndrome	☐	☐	☐	☐	☐
Having diabetes	☐	☐	☐	☐	☐
Being overweight (BMI over 25)	☐	☐	☐	☐	☐
Living a sedentary lifestyle (i.e., not being physically active)	☐	☐	☐	☐	☐
Being a smoker	☐	☐	☐	☐	☐
Taking the combined (two hormones) contraceptive pill	☐	☐	☐	☐	☐
Using tamoxifen (medication) for breast cancer treatment or prevention	☐	☐	☐	☐	☐

**12. Womb cancer diagnosis and management. Can you tell us which of the following statements are TRUE or FALSE?**			
	True	False	Don't know
Womb cancer is uncommon (i.e. less than 1 in 20 women will develop it in their lifetime)	☐	☐	☐
Womb cancer is usually diagnosed at an early stage (before it has spread)?	☐	☐	☐
Womb cancer is easily treated?	☐	☐	☐
Womb cancer has a poor prognosis (outlook)?	☐	☐	☐
Womb cancer can run in families?	☐	☐	☐

**13. NHS Cancer Screening Programmes.**	**Yes**	**No**	**Don't Know**
13a. Is there an NHS womb cancer screening programme?	☐	☐	☐
If yes, what age does screening start? ______________			
13b. Is there an NHS cervical cancer screening programme?	☐	☐	☐
If yes, what age does screening start? ______________			
13c. Is there an NHS breast cancer screening programme?	☐	☐	☐
If yes, what age does screening start? ______________			
13d. Is there an NHS bowel cancer screening programme?	☐	☐	☐
If yes, what age does screening start? ______________			

**14. How confident are you that you would notice a womb cancer symptom?**			
Not at all confident	Not very confident	Fairly confident	Confident
☐	☐	☐	☐

This is the end of the Womb CAM. Please note that this awareness measure is about womb cancer, also known as endometrial or uterine cancer.

Appendix S2

Calculation of Knowledge Scores

Warning symptoms and risk factors

When analysing responses to the unprompted items, one point was given for each warning sign or risk factor that corresponded to an item in the prompted list. Scores were totalled to give a range of 0-8 for warning symptoms and 0-10 for risk factors.

The prompted warning sign items were scored as either 0 (no/don't know) or 1 (yes). The potential score range was 0-8. Prompted risk factor items were scored from 1 (strongly disagree) to 5 (strongly agree) for the true items and 1 (strongly agree) to 5 (strongly disagree) for the protective items. This resulted in a score range of 13-65. Higher scores indicated greater womb cancer awareness.

Womb Cancer Warning Symptoms and Symptoms

Unprompted Warning symptoms and symptoms (1 point each)

- Heavy/ irregular menstrual bleeding
- Abnormal vaginal bleeding – i.e. intermenstrual or postcoital bleeding
- Postmenopausal bleeding
- Abnormal vaginal discharge
- Pelvic or back pain
- Weight loss
- Haematuria
- Anaemia

These are NOT scored as warning symptoms

- Pain during sex
- Pain on urination

Prompted warning symptoms (1 point each)

- Intermenstrual bleeding
- Heavier periods
- Postmenopausal bleeding
- Bloody discharge
- Pelvic pain
- Anaemia
- Weight loss

Incorrect

- Pain during sex

Womb Cancer Risk and Protective Factors
Unprompted risk factors

- Age or postmenopausal status
- Sedentary lifestyle (low physical activity)
- Genetics and/or family history
- Diabetes And/or Insulin resistance and/or Metabolic Syndrome
- Obesity
- Nulliparity
- Oestrogen only HRT
- High oestrogen- early menses/ late menopause
- Polycystic ovary syndrome
- Tamoxifen

These are NOT scored as risk factors

- Radiation
- Hypertension
- Hypothyroidism

Prompted Risk Factors

Score 1 to 5, 1 strongly disagree to 5 strongly agree

- Nulliparity
- Early Menarche
- Postmenopausal
- Late Menopause
- Close Relative with Womb Cancer
- Polycystic Ovary Syndrome
- Diabetes
- Overweight
- Sedentary lifestyle
- Tamoxifen use

Prompted Protective Factors

Score 1 to 5, 1 strongly agree to 5 strongly disagree

- Previous hysterectomy
- Combined oral contraceptive pill (COCP)

Age-related risk

This item was scored as 2 (correct) if a participant thought that womb cancer is most likely to develop at age 70 or 80 years, 1 (partially correct) if the response was 50 years, and 0 (incorrect) for the response '30 years old' or 'womb cancer is not related to age.' The rationale for this scoring system was that the peak age of incidence for womb cancer is 75-79, although being postmenopausal (considered over 50) increases risk.[2]

Womb cancer screening

One point was given to participants who knew that there is not an NHS womb cancer screening programme.

Total knowledge

A total knowledge score was calculated by adding the scores for items on unprompted and prompted warning symptoms and risk factors items, age-related risk, and awareness of screening (Table 1). This produced a scale where higher scores indicated greater overall awareness of womb cancer (maximum score 94).

Adaptions to scoring system for test-retest reliability analysis

The kappa statistic method used for the test-retest reliability analysis required variables to be binary. Thus, aspects of the scoring system were adapted: a participant was considered to recognise a prompted item to be a risk factor if they achieved a score greater than 4 both times when completing the Womb CAM. For the total knowledge score, it was decided that scoring 80% of the maximum score demonstrated sufficient awareness of womb cancer.

For the test-retest analysis, the kappa statistic method required variables to be binary. The prompted risk factor scoring system therefore had to be adapted so that participants were only considered to be aware of risk/protective factors if they achieved a score greater than or equal to 4, which correlated to a response of ‘agree’ or ‘strongly agree’ for risk factors, and ‘disagree’ or ‘strongly disagree’ for protective factors.
